# The strange cat scratch colon signs in a patient with multiple colon tumors

**DOI:** 10.1055/a-2432-2359

**Published:** 2024-10-16

**Authors:** Li Xu, Haijun Cao, Shanshan Chen, Jinfeng Dai, Haochen Zhang, Zemin Feng, Xuan Huang

**Affiliations:** 1 74723 Department of Gastroenterology, The First Affiliated Hospital of Zhejiang Chinese Medical University, Hangzhou, China; 2 Department of Gastroenterology, The First Affiliated Hospital of Zhejiang Chinese Medical University, Hangzhou, China

A 63-year-old man underwent a colonoscopy due to abdominal distension, during which signs of cat scratch colon were found ( Fig. 1 **a, b** ), accompanied by 10 colon tumors (3 in the cecum, 4 in the descending colon, and 3 in the sigmoid colon). Carbon dioxide was used for insufflation, and the patient did not feel discomfort during the colonoscopy. Initially, cat scratch colon was visible only in the cecum. During endoscopic mucosal resection (EMR) for colon tumors with submucosal injection (a mixture of methylene blue and physiological saline), this condition appeared again in the descending colon ( Fig. 1 **c** ) but not in the sigmoid colon ( Fig. 1 **d** ). Three months after EMR, the patient underwent a retest colonoscopy. Multiple white scars were visible in the cecum. After three minutes of observation, no signs of cat scratch colon were observed ( Fig. 1 **e, f** , Video 1 ).

**Fig. 1:**
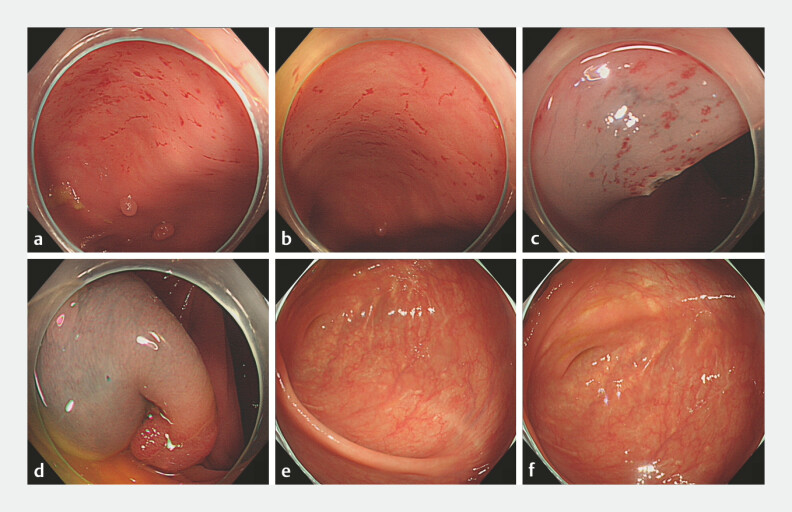
**a, b** Cat scratch colon was found in the cecum. **c** Cat scratch colon was found during endoscopic mucosal resection (EMR) in the descending colon. **d** No signs of the condition were found in the sigmoid colon during EMR. **e, f** Three months later, white scars were found instead of cat scratch colon.

Cat scratch colon was first described by McDonnell et al. in 2007 1 , and reports have been rare to date. The exact cause is not clear. At first, it was believed to be caused by air pressure injury due to insufflated air. Benjamin et al. described the first case of cat scratch colon identified using carbon dioxide insufflation in 2013 2 . Afterwards, other reasons were reported, such as collagenous colitis 3 , diversion colitis 4 , and chronic cholestasis 5 . Here, we report a case of cat scratch colon in a patient with multiple colon tumors. It is unusual that the patient did not show these signs again after EMR for the colon tumors, with white scars appearing instead. In existing case reports, this condition mainly occurs in middle-aged and elderly people, mostly aged 60 and above 1 2 3 4 5 . Therefore, we believe cat scratch colon is related to intestinal elasticity. The majority of patients experience no discomfort when experiencing colonic mucosal tears, which can heal on their own.

Cat scratch colon in a patient with multiple colon tumors. On colonoscopy three months after endoscopic mucosal resection, multiple white scars replaced signs of cat scratch colon.Video 1

Endoscopy_UCTN_Code_TTT_1AQ_2AB

## Notes

Endoscopy E-Videos https://eref.thieme.de/e-videos
*E-Videos* is an open access online section of the journal *Endoscopy* , reporting on interesting cases and new techniques in gastroenterological endoscopy. All papers include a high-quality video and are published with a Creative Commons CC-BY license. Endoscopy E-Videos qualify for HINARI discounts and waivers and eligibility is automatically checked during the submission process. We grant 100% waivers to articles whose corresponding authors are based in Group A countries and 50% waivers to those who are based in Group B countries as classified by Research4Life (see: https://www.research4life.org/access/eligibility/ ). This section has its own submission website at https://mc.manuscriptcentral.com/e-videos .

## References

[1] McDonnell WM Loura F Pointon MJ Cat scratch colon Endoscopy 2007 39 459 461 10.1055/s-2007-966266 17516354

[2] Crooks B Sampaziotis F Purkis E A dramatic finding at colonoscopy: cause for concern? Diagnosis: Cat scratch colon Gut 2013 62 1152 1213 10.1136/gutjnl-2012-303216 22982988

[3] Baudet JS Aguirre-Jaime A Factors related to the development of cat scratch colon during colonoscopy Endoscopy 2013 45 582 584 10.1055/s-0032-1326429 23780841

[4] Komuro Y Watanabe T Hata K Diversion colitis with a mucosal tear on endoscopic insufflation Gut 2003 52 1388 1389 10.1136/gut.52.9.1388 12912883 PMC1773798

[5] Purnak T Ozaslan E Yildiz A The cat scratch colon sign in a patient with chronic cholestasis Endoscopy 2010 42 E117 10.1055/s-0029-1243983 20306401

